# Mesenchymal Stem Cell-Derived Extracellular Vesicles: Regenerative Potential and Challenges

**DOI:** 10.3390/biology10030172

**Published:** 2021-02-25

**Authors:** Shivkanya Fuloria, Vetriselvan Subramaniyan, Rajiv Dahiya, Sunita Dahiya, Kalvatala Sudhakar, Usha Kumari, Kathiresan Sathasivam, Dhanalekshmi Unnikrishnan Meenakshi, Yuan Seng Wu, Mahendran Sekar, Rishabha Malviya, Amit Singh, Neeraj Kumar Fuloria

**Affiliations:** 1 Faculty of Pharmacy, AIMST University, Kedah 08100, Malaysia; 2 Faculty of Medicine, Bioscience and Nursing, MAHSA University, Kuala Lumpur 42610, Malaysia; drvetriselvan@mahsa.edu.my (V.S.); sengwu_21@yahoo.com (Y.S.W.); 3 School of Pharmacy, Faculty of Medical Sciences, The University of the West Indies, St. Augustine, Trinidad and Tobago; rajiv.dahiya@sta.uwi.edu; 4 Department of Pharmaceutical Sciences, School of Pharmacy, University of Puerto Rico, Medical Sciences Campus, San Juan, PR 00936, USA; sunita.dahiya@upr.edu; 5 School of Pharmaceutical Sciences (LIT-Pharmacy), Lovely Professional University, Jalandhar 144411, India; sudhakar.20477@lpu.co.in; 6 Faculty of Medicine, AIMST University, Kedah 08100, Malaysia; usha_harischandran@aimst.edu.my; 7 Faculty of Applied Science, AIMST University, Kedah 08100, Malaysia; skathir@aimst.edu.my; 8 College of Pharmacy, National University of Science and Technology, Muscat 130, Oman; dhanalekshmi@omc.edu.om; 9 Faculty of Pharmacy and Health Sciences, Universiti Kuala Lumpur Royal College of Medicine Perak, Ipoh 30450, Malaysia; mahendransekar@unikl.edu.my; 10 Department of Pharmacy, SMAS, Galgotias University, Greater Noida 203201, India; rishabha.malviya@galgotiasuniversity.edu.in (R.M.); amitsingh@galgotiasuniversity.edu.in (A.S.)

**Keywords:** extracellular vesicles, regenerative potential, therapeutics, cell injury, stem cells

## Abstract

**Simple Summary:**

Mesenchymal stem cell extracellular vesicles (MSCEVs) obtained from MSCs can have numerous therapeutic applications via regeneration of various body tissues. There are certain approaches by which the therapeutic effect of MSCEVs can be further potentiated. Translation of MSCEVs from the preclinical to clinical level presents several challenges to investigators. Thus, knowledge of isolation, culturing, application, and various challenges faced during clinical applications of MSCEVs are the important aspects highlighted in the present review.

**Abstract:**

Evidence suggests that stem cells exert regenerative potential via the release of extracellular vesicles. Mesenchymal stem cell extracellular vesicles (MSCEVs) offer therapeutic benefits for various pathophysiological ailments by restoring tissues. Facts suggest that MSCEV action can be potentiated by modifying the mesenchymal stem cells culturing methodology and bioengineering EVs. Limited clinical trials of MSCEVs have questioned their superiority, culturing quality, production scale-up and isolation, and administration format. Translation of preclinically successful MSCEVs into a clinical platform requires paying attention to several critical matters, such as the production technique, quantification/characterization, pharmacokinetics/targeting/transfer to the target site, and the safety profile. Keeping these issues as a priority, the present review was designed to highlight the challenges in translating preclinical MSCEV research into clinical platforms and provide evidence for the regenerative potential of MSCEVs in various conditions of the liver, kidney, heart, nervous system, bone, muscle, cartilage, and other organs/tissues.

## 1. Introduction

In the present scenario, whole-organ transplantation is considered as a major choice during treatment of end-organ dysfunction. However, the problems of scarcity of appropriate autologous tissue, chance of disease transmission, and chronic immunosuppressive treatment create the need for new therapeutic interventions [1]. The approaches of regenerative therapy and tissue engineering motivate investigators to formulate advanced strategies for damaged tissues [2]. Cellular therapies in natural or modified form are promising strategies for injured, malfunctioning, or damaged tissues. Evidence suggests that mesenchymal stem cells (MSCs) are the most suitable cell source for the engineering of injured or damaged tissue [3]. MSCs are more beneficial compared to critically differentiated cells. For example, MSCs have the potential to thwart an immune reaction and differentiate into a broad range of specific cells [4]. Isolation of MSCs can be done from various parts of the human body, such as blood, bone marrow, liver, umbilical cord, periodontal ligament, lung, and adipose tissue [5]. Despite their high regenerative potential, MSCs have been challenged in various aspects, such as clinical utility, scale-up, administration, variability, ethics, and safety [6,7,8]. 

All cells, including MSCs, secrete heterogeneous lipid bilayer vesicles called extracellular vesicles (EVs), which act as mediators for inter-cell communication. EVs play a key role in various processes, such as modulation of the immune response, homeostasis, coagulation, angiogenesis, cancer progression, and inflammation [9,10]. Based on size and origin, EVs are simply classified as small, medium, and large [11]. Growing evidence suggests that MSCs exert a beneficial effect via the release of EVs, called mesenchymal stem cell-derived extracellular vesicles (MSCEVs) [12]. Although the inability of MSCEVs to self-replicate mitigates concerns about their safety, such as uncontrolled cell division and cell contamination with tumorigenic cells [13], strict safety procedures must be followed as MSCEVs are isolated from cultured cells, and in large-scale production they would be classified as a manufactured biological product. Isolation of MSCs is often done using invasive methods, whereas MSCEV production requires in vitro culturing of MSCs. In vitro culturing allows high scalability for each MSC batch [14]. Evidence suggests that the small size of MSCEVs makes them suitable for sterilization using the filtration method [15]. MSCEVs approved at the preclinical level before entering the clinical phase face many challenges, such as production technique, quantification/characterization, pharmacokinetics/targeting/transfer, and safety profile [16]. Acknowledging these issues as major concerns, the current review presents the challenges of translating preclinical MSCEVs to the clinical level and the latest regenerative potential of MSCEVs in tissue engineering of various organs.

## 2. Biogenesis and Isolation of EVs

The biogenesis of exosomes (small size EVs ranging from 30–150 nm) is initiated from the genesis of early endosomes (from endocytoses of cell membrane), which mature into endosomes (multivesicular bodies), which accumulate intraluminal vesicles that are degraded by lysosomes and released as exosomes in the extracellular space (ECS) [17]. The biogenesis of ectosomes (medium/large size EVs ranging from 100–1000 nm) called micro-vesicles originates from direct budding of the cell membrane and release into the ECS [18]. Apoptotic bodies (large size vesicles ranging from 1–5 µm) originate from apoptotic cells [19]. Based on the cellular source and secretion mechanism, EVs hold different types of surface markers [20]. 

The tetraspanin proteins (for example, CD9, 63, and 81) are highly prevalent in exosome membranes and assist to identify exosomes. Exosomes can be differentiated based on the involvement of proteins in their genesis, such as annexin, flotillin, and auxiliary proteins (ALIX, TSG101, VPS4); constituents of endosomal sorting complex required for transport (ESCRT); heat shock proteins (HSP70 and HSP90); and GTPase enzyme [21]. CD40 and annexin A1 are related to ectosomes [22], while annexin V is related to apoptotic cells [23]. 

The biogenesis, structure, and function of EVs depend upon their key components of lipids, for example, cholesterol, sphingo-lipids (such as ceramide and sphingo-myelin), phosphor-lipid, glycerol-phospholipid, and diglyceride [24]. EVs are known to comprise nucleic acids as another important component that, after transferring to secondary cells, affects their cellular processes [9]. For example, the mRNA of some EVs substantially affects the functioning (differentiation, transcription, and proliferation) of neighbor cells. 

Various methods are employed to isolate EVs, among which ultra-centrifugation (UCF) is very common. However, UCF methods are known for their challenges regarding their need, low throughput sample, and damage to EVs (attributed to high speed) [25]. Alternative methods like gel filtration chromatography, ultrafiltration/filtration, immune precipitation, and precipitation with reagents (such as polyethylene glycol) are also used, providing different efficacy based on their quantity and purity [4]. The combination of two or more isolation methods increases EV purity [26]. The therapeutic potential of EVs can be enhanced by incorporating drugs, antibodies, proteins, and RNA for targeted delivery. Lipophilic dyes and fluorophores can also be added for in vitro/in vivo traceability. Studies recommend various novel EVs, for example, EV-based semi-synthetic vesicles, EV mimetic nanovesicles, and bioengineered EVs [27,28]. Studies have reported applications of MSCEVs for clinical complications of liver, kidney, heart, nervous system, bone, muscle, cartilage, and other organs/tissues.

## 3. Regenerative Potential (RP) of MSCEVs

### 3.1. MSCEVs and Cardiac Tissue Regeneration

Endogenous repair of damaged myocardium is usually slow and depends on the inadequate division of pre-existing cardiomyocytes and recruitment and differentiation of local cardiac stem cells [29,30]. Exogenous MSCEVs address the inadequate response to myocardial injury. Preliminary investigation recognized the ability of human embryonic-derived MSCEVs (EMSCEVs) to reduce the size of infarct in a myocardial ischemia/reperfusion injury (MI) mouse model [31] by activating the PI3K/Akt signaling pathway, which increases myocardial viability and inhibits adverse remodeling [32]. Based on this, human Akt-transfected umbilical cord-derived MSCEVs (UCMSCEVs) were developed, which exhibited enhanced endothelial cell proliferation, migration, and blood vessel formation in vivo and tube-like structures in vitro, compared to nonmodified human UCMSCEVs [33]. 

A study revealed that human amniotic fluid-derived mesenchymal stem cells (AFMSCs) and murine-induced pluripotent mesenchymal stem cells (iPMSCs) triggered cardiac regeneration via paracrine modulation of endogenous mechanisms and improved cardiac repair in an MI murine model. Administration of iPMSCEVs was shown to be safer compared to iPMSCs [34,35]. Another MI rat model study revealed that combination treatment of rat bone marrow mesenchymal stem cells (BMMSCs) and derived BMMSCEVs improved cardiac functioning, reduced infarct size, and increased neo-vascularization compared to individual treatment with BMMSCs or BMMSCEVs [36]. 

Human BMMSCs preconditioned with hypoxia improved in vitro cell biological activity [37]. The report suggests that the efficacy of BMMSCs from Cynomolgous monkeys improves when implanted for treatment of MI in monkeys [38]. Hypoxia enhanced the therapeutic effectiveness of secretory EVs, such that hypoxia conditioned human BMMSCEVs with 1% O_2_ for 3 days exhibit more cardiac regeneration in the rat MI model compared to BMMSCEVs in normoxic conditions. The report suggests that the underlining mechanism is increased angiogenesis at the infract site [39]. Hypoxia reconditioned with murine and rat BMMSCEVs (at 1% O_2_ for 72 h or 0.5% O_2_ for 24 h) inhibited apoptosis of cardiomyocytes by enriching miR-125b-5p-EV and miR-210-EV. In this case, the underline mechanism is the suppression of proapoptotic p53 and BCL2-antagonist/killer 1 (BAK1) genes and higher recruitment of cardiac progenitor cells in the infarcted heart [40,41]. MSCEVs, when encapsulated as a hydrogel, offer targeted and controlled delivery to cardiac defects. For example, a sustained release profile and higher cardiac regeneration are provided by human UCMSCEVs loaded in peptide hydrogel. The EV/hydrogel complex improves myocardial functioning via reduction of apoptosis, inflammation, and fibrosis and enhancement of angiogenesis in the infarcted boundary zone of rat hearts [42].

### 3.2. MSCEVs and Nervous Tissue Regeneration

A small injury or disease in the nervous system may lead to serious or lethal effects. The complex physiological system and limited healing capacity present the biggest challenge to nerve repair in regenerative medicine [43]. Various studies reported the repair of injured peripheral nerves (PNs) by different MSCEVs. For example, rat bone marrow MSCEVs (BMMSCEVs) regenerated injured sciatic PNs using a rat model. BMMSCEVs exhibited higher growth-associated protein (GAP43) expression, improved histomorphometric repair, and enhanced the sciatic functioning index [44]. Similarly, human umbilical cord MSCEVs (UCMSCEVs) were reported to regenerate PNs at the sites of sciatic nerve defects (SNDs) in rats. Accumulation of UCMSCEVs at the site of injured PNs stimulates axons and generates Schwann cells to fence off individual axons, reduce muscle atrophy (denervated), and modulate inflammation by upregulating anti-inflammatory cytokines (e.g., interleukin (IL)-10) and downregulating pro-inflammatory cytokines (e.g., IL1β and IL6) [45]. Rat adipose-derived MSCEVs (AMSCEVs) are also known to promote PN regeneration and neurite growth in sciatic PN defects via Schwann cell modulation [46]. The AMSCEV perinuclear location and accumulation in vesicular-like structures in Schwann cells stimulate and proliferate damaged cells, thereby indicating an endocytosis facilitated internalization pathway [47]. PN regeneration by gingiva MSCEVs (GMSCEVs) indicates proliferation and migration of Schwann cells through activation of the c-Jun N-terminal kinase (JNK) pathway and upregulation of Notch1, c-Jun, glial fibrillary acidic protein (GFAP), and Sox2 genes for de-differentiation or repair of the Schwann cell phenotype [48]. 

MSCEVs also have therapeutic potential to repair the central nervous system (CNS). Reports suggest that BMMSCEVs cause neurite development (via miR-133b transfer into neural cells) in a middle cerebral artery (MCA) stroke rat model [49]. BMMSCEV administration raised the axon density and synaptophysin-positive area (along the ischemic zone of striatum and cortex) and increased the expression of von Willebrand factor (endothelial cell marker) and doublecortin (neuroblast marker) in MCA rats [50,51], which suggest their potential for neurite remodeling, angiogenesis, and neurogenesis in stroke treatment [52]. 

BMMSCEVs, when evaluated for their potential in traumatic brain injury (TBI) using a rat model, showed improvement in the recovery of brain functioning by enhancing the counts of new immature and mature neurons in the dentate gyrus and new endothelial cells in the lesion boundary and dentate gyrus [53]. MSCEVs have also shown potential in the recovery of spinal cord injury (SCI) characterized by disrupted microvascular stability and high blood–spinal cord barrier (BSCB) permeation (due to pericyte migration) [54,55]. Mouse BMMSCEV treatment inhibited pericyte migration, thus improving BSCB structural integrity and motor functioning in an SCI rat model [56]. 

Another mechanism for SCI recovery by BMMSCEVs suggests the inhibition of neuronal apoptosis via activation of the Wnt/β-catenin signaling pathway [57]. Modification of rat BMMSCEVs with miR-133b activated the ERK1/2 and STAT3 pathways in the SCI model, which enhanced axon regeneration, neuron preservation, and locomotor functioning, compared to BMMSCEVs that were not modified with miR-133b [58]. Human placental MSCEVs (PMSCEVs) exhibited regeneration of myelin through differentiation of oligodendrocyte precursor cells (endogenous) into myelinating oligodendrocytes in vitro and enhanced myelination in the spinal cord of treated mice (multiple sclerosis model), followed by improved motor functioning [59]. Human BMMSCEVs stimulated by interferon-gamma (IFN-γ) exhibited a reduction in demyelination and neuroinflammation, thereby improving motor skills, in a multiple sclerosis experimental autoimmune encephalomyelitis (EAE) mouse model [60]. Also, in an Alzheimer disease mouse model, MSCEVs enhanced neurogenesis and cognitive function recovery [61].

Evidence suggests that neonatal hypoxic ischemic (HI) encephalopathy is one of the major reasons for newborn disability and death. MSCEVs exert a regenerative effect on HI encephalopathy; for example, a study reported the neuroprotective effect of MSCEVs in a Rice–Vannucci mouse model of severe HI-induced neonatal brain insult [62]. Another study found protective effects of MSCEVs in a preclinical model of preterm HI brain injury. The study involved in utero intravenous administration of MSCEVs into ovine fetuses with induced global HI. The study revealed that MSCEV administration improved the functioning of the brain via a reduction of seizure duration and count and preservation of the reflex sensitivity of baroreceptors [63].

### 3.3. MSCEVs and Bone Regeneration

MSCEVs provide excellent benefits in skeletal regeneration. To date, various MSCEVs have been tested for their potential in regeneration of injured bone. Human iPMSCEVs and BMMSCEVs enhanced in vitro osteogenic differentiation of BMMSCs on the one hand, and in vivo angiogenesis and bone formation in a rat model with a critical-sized calvarial defect, on the other hand [64,65]. The report suggests that dimethyl-oxaloyl glycin human BMMSCEVs stimulate angiogenesis via the Akt/mTOR pathway [66]. The efficacy of ADMSCEVs for bone regeneration can be enhanced via pre-conditioning of ADMSCs with the cytokine tumor necrosis factor alpha (TNF-α). This is based on the proliferation and osteogenic differentiation of osteoblastic cells in an in vitro experiment [67]. The study revealed that human BMMSCEV administration in a CD9 mouse model with femoral shaft fracture (with impaired bone healing ability) improved fracture healing [68]. 

In dental regeneration, administration of human dental pulp MSCEVs (DPMSCEVs) causes in vitro odontogenic differentiation. This is based on DPMSCEV endocytosis, which stimulated the P38 MAPK pathway and regenerated dental pulp-like tissue in a tooth root slice model [69]. MSCEVs are known to stimulate the migration and proliferation of periodontal ligament cells via CD73-facilitated adenosine receptor activation of pro-survival Akt and ERK signaling and periodontal regeneration in a periodontal defect rat model [70]. For improved performance of scaffold and bone healing, MSCEVs are combined with tissue-engineered constructs. For example, human ADMSCEVs are immobilized with biotin-doped polypyrrole titanium and poly (lactic-co-glycolic acid) scaffolds. In vitro investigations of BMMSCEV-based and unmodified scaffolds revealed that BMMSCEV scaffolds led to high osteoinduction of BMMSCs and osteoblasts, whereas in vivo studies with a murine model of bone defect showed improved collagen and bone tissue formation [71]. Loading of human BMMSCEVs into the tricalcium phosphate scaffold enhanced bone healing of calvarial defects via activation of the PI3K/Akt signaling pathway [72]. The report suggests that rat BMMSCEVs encapsulated in decalcified bone matrix scaffold stimulates bone regeneration by promoting vascularization in grafts [73]. 

### 3.4. MSCEVs and Liver Tissue Regeneration

Human embryonic MSCEVs are known to promote hepatic regeneration in a carbon tetrachloride (CCl4)-induced liver injury mouse model by increasing the proliferation of hepatocytes and reducing their apoptosis [74]. A report suggests that human iPMSCEVs cause regeneration of hepatic cells in a hepatic ischemia–reperfusion injury rat model via inhibition of hepatocyte apoptosis, suppression of inflammatory response, and attenuation of the oxidative stress response [75]. Human iPMSCEVs are also known to induce in vitro and in vivo proliferation of hepatocytes (in a dose-dependent manner), which is related to the activation of the sphingosine kinase and sphingosine-1-phosphate signaling pathway (which promotes cell proliferation) [76,77]. The ability of UCMSCEVs to ameliorate neutrophil infiltration and inhibit oxidative stress in hepatic tissue supports their property of protecting against hepatic apoptosis [78]. The benefits of human embryonic MSCEVs can be further enhanced by encapsulating them into PEG hydrogel, intended to sustain systemic delivery against hepatic failure. MSCEV accumulation in the liver for a prolonged period of time in a chronic hepatic fibrosis rat model had superior antiapoptotic, antifibrotic, and regenerative properties compared to conventional MSCEV administration [79].

### 3.5. MSCEVs and Kidney Regeneration

Many studies have reported the regenerative potential of MSCEVs in chronic kidney damage (CKD) and acute kidney injury (AKI). An earlier study reported that human BMMSCEVs stimulated cell proliferation, hastened the recovery of injured tubular cells, supported the functional recovery of glycerol-induced AKI, and prevented apoptosis [80]. MSCEVs exhibit their therapeutic actions using several mechanisms. One mechanism includes transportation of genetic material (mRNAs and miRNAs) to renal cells (injured), which exerts antiapoptotic, proangiogenetic, anti-inflammatory, and antifibrotic effects on AKI [81]. The second mechanism involves the parallel transfer of human IGF-1 receptor mRNA (present in MSCEVs) into tubular cells [82]. Administration of mouse and human BMMSCEVs in rat and mouse AKI models caused protection from AKI and enhanced renal functioning by stimulating tubular epithelial cell proliferation and inhibiting apoptosis [83]. The study showed that human BMMSCEVs ameliorated kidney morphology and functioning in a cisplatin-induced AKI mouse model. Based on this, an in vitro study of cisplatin-treated human tubular epithelial cells showed that BMMSCEVs upregulated antiapoptotic genes (β-cell lymphoma 2, β-cell lymphoma, extra-large and baculoviral IAP repeat-containing 8) and downregulates the genes that participate in the execution phase of cell apoptosis (caspase-1,8 and α-lymphotoxin) [84]. 

For controlled and targeted release at the site of AKI (after ischemia–reperfusion) in a mouse model, loading mouse BMMSCEVs onto self-assembling peptide nanofiber hydrogel provides a substantial increase in efficacy (improved renal function) [85]. Human UCMSCEVs are reported to induce in vitro and in vivo kidney repair in a cisplatin-induced AKI rat model by de-differentiation of tubular cells, promotion of cell proliferation, and reduction in cell apoptosis and oxidative stress [86]. Renal regeneration is also exhibited by human Wharton’s jelly MSCEVs when administered in an AKI rat model. These MSCEVs improvise renal functioning by augmenting tubular cell proliferation and reducing apoptosis and inflammation through mitochondrial fission [87]. Human glomerular and liver MSCEVs are also reported to stimulate recovery after AKI [88]. A study reported the MSCEV effect in diabetes-associated chronic kidney damage (CKD) [89]. 

Human urinary MSCEVs (UMSCEVs) are known to prevent the progression of CKD through the promotion of vascular regeneration, inhibition of podocyte apoptosis, and cell survival in a streptozotocin-induced diabetic nephropathy rat model [90]. The administration of UMSCEVs in diabetic mice showed improved renal morphology and anti-apoptotic performance of tubular epithelial cells [91]. A report suggested that human BMMSCEVs and liver MSCEVs prevent fibrosis and its progression in a diabetic nephropathy mouse model mediated by miRNA (profibrotic gene downregulator) [92]. Similar action was reported with the administration of human liver MSCEVs in an aristolochic acid-induced CKD model [93]. The Murine BMMSCEVs also exhibit protective action after renal injury in vitro and in vivo [94]. The administration of human BMMSCEVs has the potential to repair damaged mitochondria renal proximal tubule apical/basolateral membranes and improve renal functioning in a medaka model of cadmium exposure that resembles CKD on long-term exposure to heavy metals [95]. A clinical trial of 40 patients with stage III and IV CKD (n = 20 administered MSCEVs, n = 20 administered placebo) reported that UCMSCEVs provided safety and ameliorated CKD progression [96].

### 3.6. MSCEVs and Muscle Regeneration

MSCEVs also show potential in skeletal muscle regeneration. For example, human BMMSCEVs can potentially augment myogenesis and angiogenesis in vitro (mediated by miR-494) and enhance muscle regeneration [97]. Amniotic fluid MSCEVs comprise several proteins and miRNAs that can regulate inflammation and angiogenesis, boosting skeletal muscle regeneration [98]. A bioinformatics study (miRNA profiling and proteomics) that evaluated the regenerative potential of human AMSCEVs in muscle injury revealed that repair was mediated by factors that were distributed in both MSCEVs and the soluble fraction of secretomes [99].

Reports suggest that human AMSC treatment protects from muscle injury associated with torn rotator cuff. MSCEV treatment in a rat model inhibited atrophy, fatty infiltration, inflammation, and vascularization of muscles in torn rotator cuffs, and enhanced the myofiber regeneration and biomechanical properties [100]. MSCEVs derived from human urine are also known to promote the repair of pubococcygeus muscle injury in a stress urinary incontinence rat model. This is done via stimulation of extracellular regulated protein (ERP) kinase phosphorylation and activation, proliferation, and differentiation of muscle satellite cells [101]. In addition, human ASCEVs were also reported to prevent muscle damage in a mouse model of critical hindlimb ischemia via neuregulin 1 protein (NRG1)-mediated signals, which play an important role in angiogenesis, muscle protection, and prevention of inflammation [102].

### 3.7. MSCEVs and Cartilage Regeneration

Injured articular cartilage has restricted endogenous regeneration ability. Poor healing of cartilage may result in osteoarthritis (OA) [103]. A study highlighted the therapeutic action of MSCEVs on their cellular origin during OA treatment. The study compared amniotic fluid MSCs (AFMSCs) and AFMSCEVs. Animals with defects treated using AFMSCEVs exhibited higher pain tolerance and histological scores compared to AFMSCs [104]. Human BMMSCEVs stimulate in vitro regeneration of cartilage by triggering type-2 collagen and proteoglycans of chondrocyte production, which assists in cartilage repair [105]. OA is related to cartilage degradation via Wnt5A (noncanonical Wnt protein), which activates matrix metalloproteinase (MMP) and reduces cartilage ECM formation [106]. Another investigation suggests that miR92a-3p enriched human BMMSCEVs suppress degradation of cartilage and stimulate cartilage repair in an OA mouse model in vitro as well as in vivo, attributed to miR-92a-3p targeting Wnt5A [107]. Also, pre-conditioning of rat MSCs with transforming growth factor beta (TGFβ) increases the quantity of miR-135b in derived EVs, which causes stimulation of chondrocyte proliferation in vitro via specificity protein 1 (Sp1) regulation and cartilage tissue repair in an OA rat model [108]. 

A study suggests that administration of human embryonic MSCEVs in an osteochondral defect rat and mouse model caused osteochondral regeneration through well-arranged mechanisms, such as augmentation of chondrocyte proliferation, attenuation of apoptosis, and regulation of immunoreactivity at the injury site, along with formation of balance and degradation of cartilage ECM, and restoration of matrix homeostasis [109,110]. Three-dimensional culture of UCMSCs in a hollow-fiber bioreactor resulted in high yield and exceptional therapeutic potential of UCMSCEVs in a cartilage defect rabbit model compared to MSCEVs from a conventional 2D culture [111]. To retain MSCEVs at the cartilage injury site, human iPSCEVs can be incorporated in situ with hydrogel glue. Such cellular tissue patches can assimilate with the native cartilage matrix and enhance cell deposition at cartilage defect sites, thereby resulting in cartilage repair [112]. Three-dimensional printing is an advanced fabrication technique for tissue engineering that enables the development of complex forms with high precision [113]. BMMSCEVs are fabricated with cartilage ECM/gelatin methacrylate hydrogel as a bio-ink to design bio-scaffolds. The 3D-printed device enables target delivery of BMMSCEVs to prevent mitochondrial dysfunction in degenerative chondrocytes in vitro and to assist in cartilage regeneration in an osteochondral defect rabbit model in vivo [114].

### 3.8. MSCEVs and Wound Healing

The wound healing process involves complex molecular and cellular events, such as angiogenesis, cellular migration, ECM deposition, proliferation, and tissue remodeling [115]. Wounds with impaired healing fail to progress via normal healing stages (inflammation, homeostasis, proliferation, and remodeling), which leads to excessive scar formation [116]. MSCEVs exhibit beneficial effects in several chronic types of wounds. A study demonstrated that BMMSCEVs enhanced fibroblast proliferation and migration in healthy people and chronic wound patients (in a dose-dependent manner) ex vivo, and mediated tube formation by endothelial cells via activation of Akt, ERK, and STAT3 wound healing pathways [117]. An in vitro study suggested that human iPSCEVs had potential in cutaneous wound healing, by increasing collagen synthesis and angiogenesis [118]. AMSCEVs also showed the potential to increase collagen and elastin synthesis in photodamaged human dermal fibroblasts in vitro [119]. In vivo administration of AMSCEVs using a mouse skin incision model showed increased wound healing by modification of the phenotype character of fibroblasts. Collagen 1 and 3 secretion from fibroblasts increases during an early stage of wound healing, whereas collagen synthesis diminishes to reduce scar formation during the later stages [120]. A report suggested that AMSCEVs trigger keratinocyte and fibroblast migration and proliferation in an excisional wound splinting rat model via activation of the Akt pathway [121]. A comparative study of BMMSCEVs and rabbit ADMSCEVs in a rat cutaneous wound model showed significant healing [122]. Human UCMSCEVs are known to promote healing of second-degree burn wounds in vivo by activating the Wnt/β-catenin signaling pathway, increasing dermal fibroblast proliferation and angiogenesis, and reducing skin cell apoptosis [123,124]. Wound healing and suppressed scar formation are facilitated by inhibition of myofibroblast differentiation at the site of the skin defect upon treatment with human UCMSCEVs. Such effect is attributed to the activity of specific microRNAs (miR-21, 23a, 125b, and 145) [125].

### 3.9. MSCEVs and Other Tissue Regeneration

Apart from their regenerative potential in the mentioned organs and tissues, MSCEVs have regenerative potential against injuries or diseases of several other organs, such as blood vessels, esophagus, lung, and bowel. For example, human placenta MSCEVs attenuated in vitro lung cell injury (by lipopolysaccharide stimulation) [126]; swine BMMSCEVs improved in vivo lung functioning in a pig model of influenza virus-induced acute lung injury [127]; human BMMSCEVs alleviated pulmonary vascular permeability and lung injury (induced by hemorrhagic shock and trauma) in a mouse model (via activation of proteins and pathways linked to cytoskeletal rearrangement of vascular permeability) [128]; and human placenta MSCEVs inhibited calcification of synthetic vascular grafts by immunomodulation and improved vascular performance and functionality in a hyperlipidemia rat model [129].

**Figure 1:**
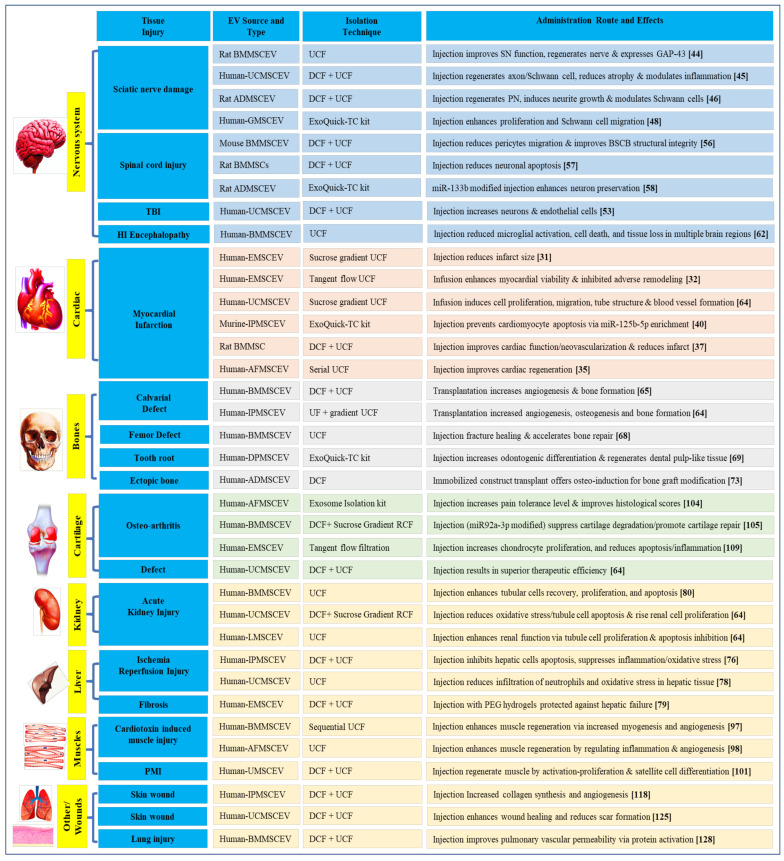
Applications of mesenchymal stem cells derived extracellular vesicles (MSCEVs) in tissue engineering.

In the same way, AMSCEVs limit abnormal proliferation and migration of vascular smooth muscle cells, and neointimal hyperplasia in vein graft bypass surgery [130]; Mouse BMMSCEVs are known to improve ulcerative colitis symptoms in a dextran sodium sulfate-induced mouse model [131]; and human UCMSCEVs ameliorate severe ischemic injury in a hindlimb ischemia mouse model [132]. AMSCEVs (inserted into thermo response hydrogels) are also known to augment healing and ensure target delivery at the site of disease in cases of tracheoesophageal diseases like fistula [133]. Similarly, numerous MSCEVs are applied in tissue engineering of various tissues and organs (Figure 1 and Table 1 show applications of MSCEVs in tissue engineering and MSCEV treatment approaches in clinical trials, respectively).

## 4. Apoptotic EVs or Apoptotic Bodies (ABs) and Their Regeneration Potential

Apoptotic cells demonstrate numerous morphological variations, such as membrane blebbing and protrusion and apoptotic body (AB) generation [134,135]. The AB membrane indicates major changes that occur on the surface of apoptotic cells. Apoptotic cells express some markers that promote their elimination by surrounding cells or macrophages before the cell membrane ruptures [136]. For example, calreticulin (the eat-me ligand) is silenced by CD47 (the do-not-eat-me ligand). Calreticulin is expressed by cells and AB only during deregulation of CD47 [137]. Characteristically AB is similar to oncosomes (EV secreted by cancer cells), however its biogenesis is different [138].

Studies have quantified the average AB production per cell as 12.87 ± 3.23 per hour [139], whereas the average production of MSCEVs by MSCs was 2900 per cell overnight [140]. Although the process of apoptosis causes the release of apoptotic microvesicles (0.1–1 μm) and small exosome-like EVs [141,142], these vesicles are less characterized compared to ABs, which are characterized based on phosphatidylserine expression and membrane permeability. ABs express phagocytosis supporting molecules (calreticulin and calnexin), chemokines and adhesion molecules (CX3CL1/fractalkine and ICAM3), and MHC class II molecules (that allow direct antigen presentation to CD4+ T cells and activate immunological memory) [143,144]. ABs may have diverse content, based on the fact that AB cargo comprises cell components that are fenced in during protrusion. ABs are known to comprise microRNAs, RNA, and DNA. The diverse contents of ABs may affect their physiological properties. ABs are further categorized into two forms: DNA-carrying and cytoplasm-carrying ABs. For DNA-carrying ABs, 5′-phosphorylated blunt-ended DNA may be used as a distinct marker. This is based on the fact that it is found exclusively in ABs, which exert apoptosis and contain DNA fragments [145].

A study suggests that AB plays an important role in immunoregulation. This is based on the quick clearance of damaged cells by immune cells (phagocytes) [144,146]. Because ABs have an important function in cell-to-cell communication among healthy and damaged cells, they are known to modulate the organ repair mechanism. Facts suggest that ABs may induce resident stem/progenitor cell proliferation, improve tissue regeneration, and replace damaged cells [139,147]. AB phagocytosis by hepatic stellate cells promotes differentiation and cell survival [148]. AB engulfing supports MSC homeostasis. Evidence suggests that systemic exogenous AB infusion could rescue apoptotic MSCs via the transfer of miR-328-3p and RNF146 and activation of the Wnt/β-catenin pathway [149].

The involvement of AB as a bioactive vehicle in cell-to-cell communication is based on a zebrafish model study, in which dying epithelial-stromal cells of epidermis produced Wnt8a-enriched ABs [139]. Neighboring p63-positive stromal cells engulfed the ABs, which activated Wnt signaling and induced cell proliferation and tissue homeostasis within 24 h via a caspase-dependent pathway. The study showed that apoptosis inhibition reduced stromal cell proliferation, whereas Wnt pathway overexpression together with apoptosis enhanced stromal cell proliferation [139].

ABs are known to release microRNA, DNA, and other genetic material into target cells, leading to different effects. For instance, the miRNA-126 of ABs induces chemokine (CXCL12) expression in healthy endothelium, and repeated administration of ABs in atherosclerotic mice produced an atheroprotective effect [150]. One study investigated whether circulating AB assists in the maintenance of MSC homeostasis and improves osteopenia by transferring multiple cellular factors. The study used a Fas-deficient MRL/lpr and Caspase 3^−/−^ mouse model to demonstrate that reduced AB biogenesis impairs the self-renewal and osteo-/adipogenic differentiation of BMMSCs. IV infusion of exogenous ABs rescued the MSC impairment and enhanced the osteopenia phenotype in MRL/lpr, caspase 3^−/−^, and ovariectomized (OVX) mice. The study determined the role of ABs in the maintenance of MSC and bone homeostasis in a pathophysiological state and suggested the potential application of ABs in the treatment of osteoporosis [149].

Another study showed that ABs derived from MSCs stimulated cutaneous wound healing by regulation of macrophage functions. The study involved extraction, characterization, application, and therapeutic evaluation of MSC-ABs in a skin wound healing mouse model. Next, AB target cells were explored, and their in vitro effects on macrophages were determined. The study demonstrated that transplanted MSCs promote cutaneous wound healing partly by releasing MSC-ABs that can convert the macrophages into an anti-inflammatory phenotype, which plays an important role in tissue repair [151]. Although the applications of ABs produced in culture as a therapeutic agent have not been tested yet, their function appears interesting in the field of regenerative medicine. Moreover, in order to establish the role of MSC-ABs in the field of regenerative medicine, numerous clinical studies are required.

## 5. Contents of MSCEVs

For a better understanding of therapeutic mechanics of MSCEVs, it is important to understand their contents. There is much evidence to suggest that MSCEVs comprise proteins, lipids, mRNAs, and miRNAs [152]. Transfer of such biochemicals or molecular factors in the target cells may influence the behavior of recipient cells [153,154]. Table 1 gives descriptions of various MSCEVs, their sources, biochemical contents, and related functions.

### 5.1. Proteins

Based on liquid chromatography–mass spectrometry (LCMS) analysis, hBMSCEVs are reported to possess 730 proteins [155]. hBMSCEV proteome function analysis determined the involvement of these proteins in cell proliferation/adhesion/migration/self-renewal, involving signaling and cell adhesion molecules, MSC-related antigens (CD9, 63, 81, 109, 151, 248, and 276), and surface receptors (Table 1). Among protein molecules, CD9, 63, and 81 are identified as typical exosomal markers [156]. MSCEVs are known to contain surface molecules (CD29, 44, 73, and 105) and tumor-supportive factors (PDGFR-β, TIMP-1 and 2) [156,157]. Human adipose tissue derived MSCEVs are also known to carry neprilysin, which causes degradation of β-amyloid peptide (both extracellularly and intracellularly) in neuroblastoma cell lines [158]. MSCEVs possess both 7α- and 7β-chains of the 20S proteasome, which reduces the deposition of denatured/misfolded proteins [159]. Evidence suggests high expression of vascular endothelial growth factor (VEGF), receptor-2 for VEGF (VEGF R2), basic fibroblast growth factor (bFGF), insulin-like growth factor I (IGF-I), Wnt4 angiogenin, monocyte chemotactic protein-1 (MCP-1), interleukin-6 (IL-6), and Tie-2/TEK proteins in human umbilical cord derived MSCEVs. These proteins participate in the promotion of β-catenin nuclear translocation and enhancement of the angiogenesis process [160,161]. A study suggests that MSCEVs contain ribonucleoproteins, namely, AU-rich element-binding protein (Hu R), staufen2 (Stau2), argonaute2 (Ago2), staufen1 (Stau1), T cell internal antigen-1 (TIA), and TIA-1-related (TIAR) proteins. These proteins are responsible for mRNA transportation and stability [162].

### 5.2. Nucleic Acids

Nucleic acids, including mRNAs and miRNAs, are another important component of MSCEVs [152]. MSCEVs transfuse into the membranes of target cells and transfer mRNAs and miRNAs into recipient cells located in the tumor micro-environment or remote regions.

#### 5.2.1. mRNA

mRNAs present in EVs are connected with mesenchymal phenotypes and numerous cell functions associated with regulation of cell differentiation (OR11H12, GRIN3A, RAX2, DDN, and OR2M3), cell transcription (IRF6, CLOCK, BCL6B, RAX2, and TCFP2), cell proliferation (RBL1, SENP2, S100A13, and CDC14B), cytoskeleton (CTNNA1, DDN, and MSN), cell metabolism (FUT3, ADAM15, LTA4H, ADM2, RAB5A, and BDH2) [47], and cell immune regulation (CRLF1, IL1RN, and MT1X) (Table 2). An in vitro toxic renal injury model showed that MSCEVs transferred insulin growth factor 1 (IGF-1) mRNAs, which caused proximal tubular cell proliferation [163]. High-throughput RNA sequencing revealed selective expression of distinctive RNAs in porcine adipose tissue-derived MSCEVs [164]. Generally, MSCEVs express mRNAs for adipogenesis, Golgi apparatus, angiogenesis, and transcription factors related to the organization of chromosomes, alternative apoptosis, and splicing. Apart from that, MSCEVs are also reported to express the genes for TGF-β signaling (FURIN, ENG, and TGFB1 and B3).

#### 5.2.2. microRNA (miRNA)

MSCEVs are also shown to possess microRNAs. miRNAs are small noncoding RNAs that possess 22 nucleotides [172]. After internalization through target cells, miRNAs play an important role in tumor suppression and targeting of specific mRNAs to inhibit translation [173]. A study using an miRNA array detected 79 mature miRNAs in BMSCEVs [167]. Among these, five miRNAs (miRNA-199b, 218, 148a, 135b, and 221) were expressed at different time intervals in BMSCEVs during osteogenic differentiation. Studies were also done to analyze the miRNA profile of MSCEVs released by adipose-derived and bone marrow-derived MSCs (AMSCs and BMSCs). MSCEVs are primarily composed of mature transcripts. The majority of miRNAs expressed in AMSCEVs and BMSCEVs are similar but differ in proportion. So, it may be possible that AMSCEVs and BMSCEVs transfer distinct information [171,174]. Human embryonic MSCEVs were enriched with precursor miRNAs compared to mature miRNAs [175]. Therefore, MSCEVs released from various MSC sources might comprise different types of miRNAs. MSCEVs are also known to possess other miRNAs (miRNA-15a, 16, 21, 34a, and 191) [156,168,170]. These miRNAs mediate the promotion of cellular growth, reduction of cardiac fibrosis, prevention of apoptosis, and inhibition of tumor growth through the regulation of target genes in recipient cells [176,177]. If the miRNAs are not randomly sorted in MSCEVs, some miRNAs may be present in the original cells only, but not in the MSCEVs. Some miRNAs are sorted selectively into MSCEVs, which cannot be detected in the original MSCs (for example, miRNA-564, 223, and 451). The specific mechanism of MSCEV content sorting is still unclear.

#### 5.2.3. Lipids and Other Contents

Studies have shown that MSCEVs comprise a high concentration of bioactive lipids, namely, diacylglycerol (DAG) and sphingomyelin (SM), along with trace quantities of dihydroceramide (DHC) and α-hydroxy-ceramide (AHC). Apart from that, small molecule metabolite assays showed the presence of glutamic acid and lactic acid in MSCEVs [157].

## 6. Challenges for MSCEVs

Preclinically approved MSCEVs, when subjected to clinical experiments, involve several challenges based on the following aspects.

### 6.1. Mass Production of MSCEVs

The traditional methods of assisted massive scale production of MSCEVs from MSCs for longer duration may leave out the cloning and differentiation properties of MSCs [178]. This necessitates the advancement of traditional approaches for the mass production of MSCEVs. The traditional methods of mass producing MSCEVs from MSCs are laborious, and include cell culturing in small flasks or 2D culturing in bioreactors [178,179,180]. Generally, the traditional methods for producing MSCEVs provide lower yield and cannot be scaled up, thereby limiting the clinical application of MSCEVs as therapeutics [181]. MSCEVs can be produced on a massive scale by using culturing flasks (big or multi-layered), bioreactors (fixed-bed or in-stirred tank), and perfusion reactors (continuous production) [182]. These methods improve production by increasing the culture surface area compared to conventional planar MSC culturing in flasks [183]. One study reported a 20 times higher yield of MSCEVs from hUCMSCs using scalable microcarrier-based 3D culturing compared to 2D culturing [111]. To assure production consistency and reproducibility for MSCEV extraction using cell culturing supernatants, the technical factors must be controlled [184].

There are several key factors that may affect the quality and quantity of MSCEVs produced from MSCs, such as cellular confluence, early versus later passage of cells, oxygen concentration, cytokines, heparin, and medium serum content [185]. Fetal bovine serum (FBS), which serves as a nutrient medium for the growth of MSCs in culture, contains RNA-bearing EVs that may affect the behavior of the MSC culture. This necessitates the development of an experimental protocol to eliminate such interference during the production of MSCEVs [186]. A study reported that serum-free culture affected the composition of proteins and quantity of EVs [187]. To resolve such a problem, culturing human bone-marrow derived MSCs in serum-free media (that is, EVs depleted with a low level of human platelet lysate) offers a protocol (compliant with good manufacturing practice) wherein it is possible to produce MSCs and MSCEVs on a large scale. Use of this protocol showed that hBMMSCs were affected in terms of proliferation and differentiation ability; retained morphology, viability, differential potential, and phenotypes; and enhanced hBMMSC–EVs [188]. The yield of MSCEVs can also be enhanced by manipulating the biology of EV biogenesis [189].

### 6.2. Scalable Methods for MSCEV Isolation

Compared to mass production, scalable methods for MSCEV isolation are difficult when used for clinical translation in larger quantities. MSCEV isolation can be done using different methods [190,191]. Large-scale isolation of EVs involves differential centrifugation (DCF), size-exclusion chromatography (SEC), ultracentrifugation (UCF), immuno-based capturing (IBC), and precipitation methods [182]. Studies suggest the use of ultrafiltration and size exclusion chromatography mediated isolation of EVs (from SC culture) in high yield with preserved functional and biophysical properties [192,193,194]. Compared to affinity-based isolation of EVs from human plasma, size exclusion chromatography-based isolation of EVs is reported to be superior in biomarker and therapeutics research [195]. Size exclusion chromatography alone cannot separate the EVs in plasma from lipoproteins [196].

### 6.3. Stability

After mass purification, stability is an important factor to consider in clinical applications of MSCEVs. To assure stability, MSCEVs need a suitable environment for storage. Commonly collected pure MSCEVs are resuspended in PBS and stored at −80 °C [197,198]. Interestingly a study suggests that trehalose may enhance MSCEV stability. Trehalose is known to stabilize proteins, phospholipid bilayers, and dry membranes [199]. The study suggested that trehalose limits the aggregation and fusion of β-cell exosome-like vesicles (β-ELVs). It was shown that with repeated freezing and thawing, β-ELV integrity and bioactivity were better when the β-ELVs were stored in trehalose compared to PBS. The study proved that trehalose is a good cryoprotectant for cryogenic storage of clinical-grade MSCEVs [200]. Though storage of MSCEV at −80 °C is a good option, it may come with cost limitation and transportation difficulty. An investigation explored MSCEV storage using the lyophilization method, which involved trehalose to protect EVs from osmotic damage (during lyophilization) and storing samples at 25 °C (after lyophilization). The results showed that lyophilization had little effect on exosomes, including the physical and biological characteristics [201]. Due to the complex nature of the MSCEV production process, for each type of MSCEV it is very important to explore different isolation methods and optimization of best storage conditions supported by more research evidence over a variety of cell sources.

### 6.4. MSC-EV Biodistribution and Tissue Targeting

Understanding the therapeutic properties of MSCEVs requires insight on MSCEV biodistribution and targeting mechanics. It is possible to study various labeled tissue targets using noninvasive optical imaging methods, such as near-infrared dyes, which amplify tissue penetration [202,203,204]. A study reported labeling of MSCEVs in an acute kidney injury mouse model by direct labeling of EVs and labeled EV production from near-infrared (NIR) dyed pre-incubated MSCs. MSCEVs were detectable in whole-body images using optical imaging. The NIR dye-labeled EVs exhibited high fluorescence compared to the labeled MSCEVs. Receptor-mediated interactions assist in the recruitment of MSCs at the injured site [205]. MSCEVs that have the same MSC membrane receptors may also be recruited to the injured site via the same mechanism [203].

The biodistribution of MSCEVs can be traced with different dyes. For example, DiD lipid dye-labeled MSCEVs were used to image MSCEV distribution in mice. The labeled MSCEVs exhibited maximum distribution in spleen and liver, less in bone marrow (femur, spine, tibia), and none in kidney, heart, and lungs [204]. MSCEVs can also be labeled using PKH-26A (lipophilic dye), which integrates into cell membranes [46,206,207]. Rat adipose-derived MSCEVs exhibited sciatic nerve regeneration and neurite growth. The derived MSCEVs increased the regeneration of injured sciatic nerve and neurites [46]. A study using a carotid artery balloon injury rat model suggested DIO labeled MSCEVs as a therapeutic target in vascular disorders. This was based on the ability of MSCEV miR-125b to transfer to vascular smooth muscle and attenuate neointimal formation [208]. Reports suggest the use of gadolinium, Alexa Fluor 488, and DiI in MSCEV labeling to determine the biodistribution of EVs [158,159,160].

A preclinical study using a rat stroke model highlighted PKH26 and carboxyfluorescein diacetate succinimidyl ester (CFSE) based labeling to determine the biodistribution, therapeutic action, and mechanism of MSCEVs [209]. The study revealed that migration and accumulation of MSCEVs into the infarcted brain was dose-dependent. The administered MSCs at a high dose accumulated in the lung and liver, which suggests that MSCs seldom target tissues [210]. The therapeutic mechanism of MSCEVs is still unknown; however, MSCEV cargo may involve mRNAs, microRNAs, and membrane/cytoplasmic proteins. A study suggests that the therapeutic action of MSCEVs is via the transfer of miRNAs into injured cells [211]. The miRNAs of MSCEVs are reported to affect pathophysiological microenvironments and mediate cardiac protection and regeneration [209,212]. The approaches to load and modify MSCEV cargo include electroporation, freeze-thaw cycles, hypotonic dialysis, and saponin-assisted loading [213,214]. The MSCEV cargo affects the migration of MSCEVs. MSCEVs express chemokine receptors, which assist in targeting injured tissue regions [209,215]. It has been suggested that modification of the MSCEV surface with phosphatidylserine and HER2-targeting proteins increases MSCEV distribution to HER2-expression cells [216]. Several investigations highlight modification approaches to influence EVs to target specific tissues [217], so these approaches may also be applied to MSCEVs. Although the specific mechanism of MSCEVs is unknown, it is hypothesized that they act in the same way as MSCs (which provide therapeutic action by secreting factors that reduce cellular injury and promote repair). MSCEVs may act as communication vehicles to support signals of the tissue microenvironment [218,219].

### 6.5. Heterogeneity

The heterogeneity of MSCs is another challenge that affects the clinical translation of MSCEVs. The size, molecular content, and biological role of MSCEVs are cell-type dependent and may vary even if the MSCEVs are derived from the same parent cells. A study revealed that based on their size and CD44 content, breast cancer cells may release various forms of exosomes [220]. Biomolecular factors such as the pathophysiological state of the parental cells, extracellular stimulus, and biogenetic pathways may lead to heterogeneity of MSCEVs [221]. Based on the fact that miRNAs get packed in EVs via different mechanisms, the miRNAs of MSCEVs may vary even when they are derived from the same tumor, whereas some miRNAs may be expressed in most MSCEVs, and apart from that, miRNAs are enriched only in specific subclasses of EVs [222]. Evidence suggests that in MSCEVs, the miRNAs exist as a blend of highly expressed cellular miRNAs (that are incorporated in MSCEVs through an osmosis kind of effect) and selectively secreted miRNAs (that are packed in MSCEVs depending on the specific RNA sequence) [223]. A study determined that more than 65% of released miRNAs are secreted passively through MSCEVs based on the quantity of cytoplasmic miRNA, whereas 30% of miRNAs of MSCEVs do not represent the cell profile. This shows that miRNAs are released selectively [224].

Like the miRNA content, the protein content of MSCEVs also exhibit similar features. For example, a study identified two subpopulations of EVs (LD-Exo and HD-Exo) from B16F10 melanoma cells through sucrose density gradient centrifugation. Both wrapped the same proteins, Alix and TSG101. HD-Exo contained protein ephrin type-A receptor 2, whereas LD-Exo contained actinin α4 and cyclin Y. The study determined that the relative abundance of the same protein was not common [225]. Moreover, MSCEVs from the apical and basolateral regions of some cells also exhibited variation, leading to promotion and maintenance of cell polarization [226]. Rab27a was reported to inhibit this effect (by reducing EV secretion through ceramide biosynthesis degradation) and reduce EV secretion of (by regulation of intracellular compartments); however, about 30–50 nm EVs remained the same. This indicates different origins of MSCEVs, except for intracellular compartments (plasma membrane) [227]. MSCEV secretion also relies upon ESCRT-dependent or -independent sorting pathways, including various molecules such as tetraspanins, which partly explains different subtypes of MSCEVs [228]. The majority of studies use MSCEVs as bulk isolates while evaluating their efficacy. The difficulty in separating specific MSCEVs includes the scarcity of unique molecules to distinguish each MSCEV subtype and specific isolation methods. Hence, there is an urgent need to advance the technology in order to address this problem. This will assist in gaining a better understanding of the heterogeneity of MSCEVs and hasten the development of MSCEV-based therapeutics.

### 6.6. Safety Profile

**Figure 2:**
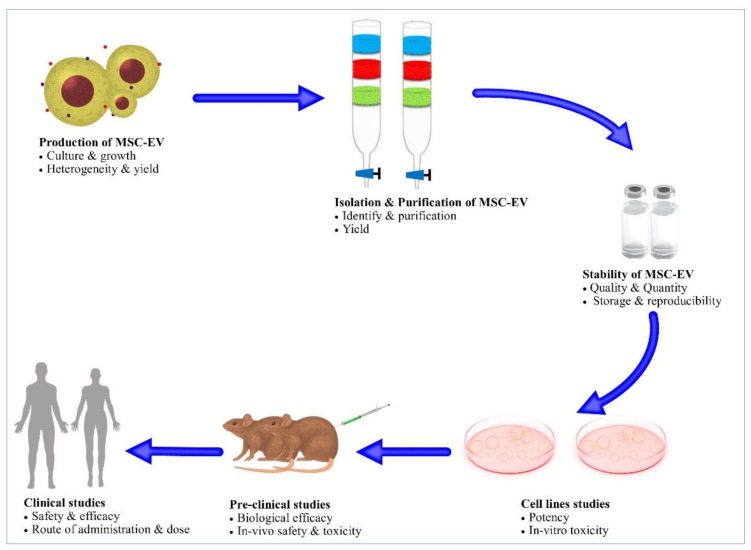
Illustration of various challenges associated with clinical development of MSCEVs.

Based on their high biocompatibility, low toxicity, and immunogenicity, MSCEVs are considered to be excellent delivery vehicles. MSCEVs are already tested at various levels of preclinical and clinical research. Numerous studies have reported MSCEV tolerance, whereas only a few studies have examined security issues. However, a study was done on the hepatotoxicity and immunogenicity of MSCEVs [229]. The study revealed that with high exposure of HepG2 cells to EV cells (derived from Expi293F), there was no significant structural or functional effect. The study reported that EVs mediated no incidence of inflammation in recipient cells. Evidence suggests no incidence of toxicity and immune response by EVs at high doses in immune-intact mice. Tumor cell-derived EVs also had no impact on oncogenic or DNA damage pathways of HepG2 cells. Intraperitoneal (IP) and intravenous (IV) administration of HEK293T-derived EVs (loaded with miR-199a-3p and chimeric proteins) for 3 weeks exhibited no significant toxicity or immune response [230]. The differentiation of MSCs and their potential to suppress the anti-cancer immune response and act as a progenitor for blood vessels can potentially promote tumor growth and metastasis [231]. MSCs are impeded based on immunogenicity, genomic mutability, and tumorigenicity; however, MSCEVs do not have such shortcomings [17,232]. Some clinical studies involving simple EVs (from non-MSC sources) reported good safety profiles [233]. As MSCEVs lack features to cause the mentioned issues, this supports their importance as therapeutic agents. An illustration of various challenges associated with clinical development of MSCEVs for the detection of CSCs is given in Figure 2. The benefits associated with MSCEVs suggest their clinical application in place of MSCs in the future.

## 7. Conclusions

The therapeutic efficacy of EVs from different tissues against various diseases and injuries opens up a wide range of opportunities in tissue engineering and regenerative medicine. However, limited clinical studies are available on the enhancement of the beneficial effects of MSCEVs along with bio-engineering and genetic modification, nanomaterials, and drug encapsulation. Therefore, developing MSCEVs for clinical use presents several challenges to investigators and clinicians. The major challenges are to establish optimum reliability, reproducibility, and robust techniques to isolate and purify therapeutic EVs and to produce EVs on a large scale with good manufacturing practices standards for clinical use. The future of MSCEVs depends upon standardized large-scale MSC culturing to establish them as a successful therapeutic choice. MSCEVs in hypoxic conditions and treatment with miRNAs increases MSC growth and MSCEV release substantially. Substantial developments have been made in the isolation and characterization of MSCEVs. Size exclusion chromatography has the ability to provide specific MSCEV substituted standard approaches of density-gradient centrifugation and immunoprecipitation. In therapeutic research, the specific size of MSCEVs is a major concern. Size exclusion chromatography permits more specific extraction of MSCEVs, assuring treatment specificity and efficiency. MSCEV biodistribution and targeting are inadequately identified; however, MSCEVs are important messengers between MSCs and injured tissues, such that when MSCs accumulate in inadvertent tissues, their secreted MSCEVs target the injured tissues. An in-depth understanding of potential actions of MSCEVs requires further understanding of their targeting and biodistribution. Establishing a therapeutically beneficial sub-population of heterogeneous EVs is another challenge, as clear classification of different subtypes is still under investigation. At present, several studies highlight the therapeutic efficacy of exosomes in the engineering of various body tissues and organs like the brain, kidney, liver, bones, cartilage, lungs, muscles, bowel, esophagus, and blood vessels. However, further research is warranted to establish suitable therapeutic doses and routes of administration for the clinical use of EVs in the future.

## Figures and Tables

**Table 1:** Mesenchymal stem cell extracellular vesicle (MSCEV) treatment approaches in clinical trials. AMSCEV, adipose-derived MSCEV; BMMSCEV, bone marrow-derived MSCEV; UMSCEV, urine-derived MSCEV; CKD, chronic kidney disease.

Condition	Injured/Diseased Tissue	Treatment Approach	Trial Phase
Healthy	Injured lungs	Aerosol-based inhalation of AMSCEV (1 or 2 or 3 × 10^8^ particles/3 mL)	I [64]
Osteoarthritis	Injured cartilage	Osteochondral explant from arthroplasty treatment with AMSCEVs	
Bronchopulmonary dysplasia	Chronic lung disease	Intravenous infusion of BMMSCEVs (20 or 60 or 200 pmol phospholid/kg body weight)	
COVID-19	Lungs (pneumonia)	Aerosol inhalation of AMSCEVs (2 × 10^8^ particles/3 mL) for 5 days	
Dystrophic epidermolysis bullosa	Skin	Local administration of allogenic BMMSCEVs	II [64]
Acute ischemic stroke	Injured brain	Stereotaxis-based administration of miR-124 (200 µg total EV protein) enriched MSCEVs	
Kidney	CKD	Injection of allogenic UMSCEVs (100 µg total EV protein/kg/dose)	II/III [96]

**Table 2:** Components and roles of MSCEVs from different MSC sources.

MSCEV Components	Source	Biochemical Factors/Genes	Functions	References
Proteins	hAMSC	Neprilysin	Degradation of *β*-amyloid peptide (intracellular and extracellular) in neuroblastoma cell lines	[158]
	hBMSC	EGFR, PDGFRB, IGF2R and TGFBI	Self-renewal of MSCs	[165]
	hBMSC	PRKACA, CTNNB1, PPP2R1A, CHP, PRKCB, CAMK2D and 2G, and RAC1 and 2	Wnt signaling, self-renewal and differentiation of MSCs	[165]
	hBMSC	CD-9, 13, 29, 44, 63, 73, 81, 90, and 105	Surface antigen	[157,165]
	hBMSC	PPP2R1A, CD105, ENG, USP9X, COL1A2, and MAPK1	TGFβ signaling and differentiation of MSCs	[165]
	hBMSC	CDC42 and 81; RAC1 and 2; FLNA, B, and C; HSPAB, Bl, and A1A; RAP1A and B; PRKCB and ACA; CACNA2D1; CHP; PDGFRB; RRAS2; MAP4K4; NRAS; CAVI; PPP2RIA; EGFR; RRAS; GNG12; MAPKl; RAP1A; GNAI2; PRDX2; LPAR1; ITGA1; and SOD1	PPAR signaling and differentiation of MSCs	[165]
	hBMSC	ILK, ACSL4, and FABP5	PPAR signaling and differentiation of MSCs	[165]
	hBMSC	ENG, USP9X	BMP signaling and differentiation of MSCs	[165]
	hBMSC	HuR, TIA, and TIAR	T cell internal antigen	[162]
	hBMSC	Stau1 and Stau2	mRNA transportation and stability	[162]
	hBMSC	Ago2	Assists in transportation and processing of miRNAs	[162]
	hUMSC	IL6, MCP1, IGFI, UPAR, bFGF, VEGF, VEGFR2, and angiogenin	Promotes angiogenesis	[123,161]
	hUMSC	Wnt4	Enhances proliferation and migration	[162]
mRNA	hBMSC	IGF-1R	Improves proliferation of cells	[82]
	hBMSC	OR11H12, RAX2, OR2M3, GRIN3A, DDN, NIN, IBSP, BMP15, MAGED2, HK3, EPX, COL4A2, PKD2L2, CEACAM5, and SCNN1G	Mediates cell differentiation	[80]
	hBMSC	IRF6, CLOCK, RAX2, BCL6B, and TCFP2	Mediates transcription	[80]
	hBMSC	TOPORS, HMGN4, ELP4, ESF1, HNRPH2, and POLR2E	DNA/RNA binding	[80]
	hBMSC	RBL1, SENP2, S100A13, and CDC14B	Cell cycle	[80]
	hBMSC	CXCR7, CEACAM5, and CLEC2A	Receptors	[80]
	hBMSC	FUT3, ADAM15, ADM2, BDH2, RAB5A, and LTA4H	Mediates metabolism	[80]
	hBMSC	MT1X, CRLF1, and IL1RN	Immune regulation	[80]
	hBMSC	CTNNA1, DDN and MSN	Cytoskeleton	[80]
	hBMSC	IBSP and COL4A2	Extracellular matrix	[80]
	pAMSC	KDM6B, JMJD1C, and FOXP3	Encodes transcription factors of chromosome organization	[164]
	pAMSC	IFT57, MDM4, PDCD4 and PEG3	Encodes transcription factors of apoptosis	[164]
	pAMSC	HES1, TCF4 and HGF	Encodes transcription factors of proangiogenic pathways	[164]
	pAMSC	ZHX1; ZBTB1; and ZNF217, 238, 568, 461, and 667	Encodes zinc-finger transcription factors	[164]
	pAMSC	BAZ2B, TMF1, JMJD1C, NFKBIZ, PEG3, MYNN, KCNH6, SUFU, and RUNX1T1	Encodes transcription factors related to alternative splicing	[164]
miRNA	ratBMSC	miRNA-133b	Contributes to neurite outgrowth	[166]
	hBMSC	miRNA-148a, 135b, 199b, 218, and 221	Regulates differentiation of osteoblasts	[167]
	hBMSC	miRNA-15a	Inhibits multiple myeloma cell growth	[168]
	pAMSC	miRNA-148a and 378, let-7f, and miR532-5p	Regulates apoptosis, proteolysis angiogenesis, and cellular transport	[164]
	hBMSC	miRNA-21 and 34a	Regulates cell survival and proliferation	[156]
	hBMSC	miRNA-23b	Induces dormant phenotypes	[169]
	hBMSC	miRNA-16	Targets VEGF and suppresses angiogenesis	[170]
	hAMSC	miRNA-10a-5p, 10b-5p, 21-5p, 22-3p, 26a-5p, 51a-3p, 92a-3p, 92b-3p, 99b-5p, 100-5p, 127-3p, 143-3p, 146a-5p, 146b-5p, 191-5p, 222-3p, 486-5p, 4485; and let-7a-5p, and 7f-5p	Mediates replicative senescence and immunomodulation; regulates cell cycle progression and proliferation; modulates angiogenesis; promotes migration	[171]
	hBMSC	miRNA-10a-5p, 10b-5p, 21-5p, 22-3p, 27b-3p, 28-3p, 92a-3p, 92b-3p, 99b-5p, 100-5p, 125b-5p, 127-3p, 143-3p, 191-5p, 222-3p, 423-5p, 486-5p; let-7a-5p, 7f-5p, and 7i-5p	Assists ASC replicative senescence, immune modulatory function; promotes migration; regulates cell cycle progression and proliferation; modulates angiogenesis	[171]

## Data Availability

Data is contained within the article.

## Abbreviations

ACLS4	Acyl-CoA synthetase long chain family member 4
ADAm	A disintegrin and metalloprotease
ADM2	Adrenomedullin 2
AFMSCEV	Amniotic fluid-derived MSCEV
AGO2	Argonaute RISC catalytic component 2
AKI	Acute kidney injury
Akt	Serine/threonine kinase (also called as protein kinase B)
ALIX	ALG-2-interacting protein X
AMSCEV	Adipose-derived MSCEV
BAK1	BCL2-antagonist/killer 1
BAZ2B	Bromodomain Adjacent to Zinc Finger Domain 2B
BCL6B	B-cell CLL/lymphoma 6 member B protein
BDH2	3-hydroxybutyrate dehydrogenase type 2
bFGF	Basic fibroblast growth factor
BMMSCEV	Bone marrow-derived MSCEV
BMP15	Bone morphogenetic protein 15
BSCB	Extracellular signal-regulated kinase
CACNA2D1	Calcium voltage-gated channel auxiliary subunit alpha 2 delta 1
CAMK2D	Calcium/calmodulin-dependent protein kinase type II delta
CAVI	Carbonic anhydrase VI
CCl4	Carbon tetra chloride
CD	Cluster of differentiation
CDC42	Cell division control protein 42
CEACAM5	Carcinoembryonic antigen-related cell adhesion molecule 5
CKD	Chronic kidney damage
CLEC2A	C-type lectin domain family 2 member A
CLOCK	Circadian locomotor output cycles kaput
CNS	Central nervous system
COL1A2	Collagen type I alpha 2 chain
CRLF1	Cytokine receptor-like factor 1
CTNNA1	Catenin Alpha 1
CTNNB1	Catenin Beta 1
CXCR7	C-X-C chemokine receptor type 7
2D	2 Dimensional
3D	3 Dimensional
DCF	Differential centrifugation
DDN	Dendrin
DPMSCEV	Dental pulp MSCEV
EAE	Experimental autoimmune Encephalomyelitis
ECM	Cartilage extracellular matrix
ECS	Extracellular space
EGFR	Epidermal growth factor receptor
ELP4	Elongator acetyltransferase complex subunit 4
EMSCEV	Embryonic-derived MSCEV
ENG	Endoglin
EPX	Eosinophil Peroxidase
ESCRT	Endosomal sorting complex required for
EV	Extra cellular vesicles
FABP5	Fatty acid binding protein 5
FLNA	Filamin A
FOXP3	Forkhead Box P3
FUT3	Fucosyltransferase 3
GAP43	Growth Associated Protein 43
GFAP	Glial fibrillary acidic protein
GMSCEV	Gingiva MSCEV
GNAI2	Guanine nucleotide-binding protein G(i) subunit alpha-2
GNG12	G protein subunit gamma 12
GRIN3A	Glutamate ionotropic receptor NMDA type subunit 3As
GTP	Guanosine triphosphate
hAMSC	Human adipose tissue-derived MSC
hBMSC	Human bone marrow-derived MSC
HES1	Hairy and enhancer of split-1
HGF	Hepatocyte growth factor
HK3	Hexokinase 3
HMGN4	High mobility group nucleosomal binding domain 4
HNRPH2	Heterogeneous nuclear ribonucleoprotein H2
HSP	Heat shock protein
hUMSC	Human umbilical-derived MSC
HuR	Human antigen R
IAP	Inhibitor of apoptosis
IBSP	Bone sialoprotein
IFN	Interferon-gamma
IFT57	Intraflagellar transport 57
IGF-1	Insulin-like growth factor 1
IGF2R	Insulin-like growth factor 2 receptor
IL	Interleukin
ILK	Integrin Linked Kinase
IL1RN	Interleukin 1 receptor antagonist
iPMSCEV	induced pluripotent MSCEV
IRF6	Interferon regulatory factor 6
ITGA1	Integrin alpha-1
JMJD1C	Jumonji domain containing 1C
JNK	c-Jun N-terminal Kinase
KCNH6	Potassium voltage-gated channel subfamily H member 6
KDM6B	Lysine-specific demethylase 6B
LPAR1	Lysophosphatidic acid receptor 1
LTA4H	Leukotriene A4 hydrolase
MAGED2	Melanoma-associated antigen D2
MAPK	Mitogen-activated protein kinase
MCA	Middle cerebral artery stroke
miR	Micro RNA
MMP	Matrix metallo proteinase
mRNA	Messenger ribose nucleic acid
MSC	Mesenchymal stem cells
MSCEV	Mesenchymal stem cell-derived extracellular vesicles
MSN	Moesin
MT1X	Metallothionein 1X
mTOR	Mammalian target of rapamycin
MYNN	Myoneurin
NIN	Ninein
NKFBIZ	NFKB Inhibitor Zeta
NRAS	Neuroblastoma RAS
O2	Oxygen
OA	Osteo arthritis
OR11H12	Olfactory receptor family 11 subfamily H member 12
OR2M3	Olfactory receptor 2M3
pAMSC	Pig adipose tissue derived mesenchymal stem cells
PCP1	Monocyte chemoattractant protein-1
PDCD4	Programmed cell death 4
PDGFRB	Platelet-derived growth factor receptor beta
PEG	Polyethylene glycol
PEG3	Paternally-expressed gene 3
PI3	Phosphatidylinositol 3
PKD2L2	Polycystic kidney disease 2-like 2 protein
PN	Peripheral nerve
POLR2E	RNA polymerase II, I and III subunit E
PPR2R1A	Protein phosphatase 2, regulatory subunit A
PRAKACA	Protein kinase A - catalytic subunit
PRDX2	Peroxiredoxin-2
PRKCB	Protein Kinase C Beta
RAC1	Ras-related C3 botulinum toxin substrate 1
RAP	Ras-related protein
RAP1A	Ras-related protein Rap-1A
ratBMSC	Rat bone marrow-derived mesenchymal stem cells
RBL1	RB transcriptional corepressor like 1
RP	Regeneration potential
RRAS	Ras-related protein R-Ras
RUNX1T1	RUNX1 partner transcriptional co-repressor 1
S100A13	S100 calcium binding protein A13
SCI	Spinal cord injury
SCNN1G	Sodium channel epithelial 1 subunit gamma
SENP2	Sentrin-specific protease 2
SND	Sciatic nerve defect
SOD1	Superoxide dismutase type 1
STAT	Signal transducer and activator of transcription
Stau	Staufen double-stranded RNA binding protein
SUFU	Suppressor of fused protein
TBI	Traumatic brain injury
TCF4	Transcription factor 4
TCPF2	Transcription factor CP2
TGFB1	Transforming growth factor beta induced
TIA	T-cell intracellular antigen
TMF1	TATA element modulatory factor
TNF	Tumor necrosis factor
TOPORS	Topoisomerase I-binding RS protein
TSG	Tumor susceptibility gene 101
UCF	Ultracentrifugation
UCMSCEV	Umbilical-derived MSCEV
UMSCEV	Urinary MSCEV
UPAR	Urokinase plasminogen activator surface receptor
USP9X	Ubiquitin specific peptidase 9 X-linked
VEGF	Vascular endothelial growth factor
VPS	Vacuolar protein sorting-associated protein transport
Wnt	Wingless-related integration site
XHX1	Zinc fingers and homeoboxes 1
ZBTB1	Zinc finger and BTB domain containing 1
ZNF217	Zinc finger protein 217
